# Direct access to potential research participants for a cohort study using a confidentiality waiver included in UK National Health Service legal statutes

**DOI:** 10.1136/bmjopen-2016-011847

**Published:** 2016-08-01

**Authors:** Rachel M Taylor, Lorna A Fern, Natasha Aslam, Jeremy S Whelan

**Affiliations:** Cancer Division, University College London Hospitals NHS Foundation Trust, London, UK

**Keywords:** Trial recruitment, BRIGHTLIGHT, Alternative recruitment methods

## Abstract

**Objectives:**

To describe our experience of using a confidentiality waiver (Section 251) in the National Health Service (NHS) Act to identify and recruit potential research participants to a cohort study and consider its use in a wider research context.

**Design:**

Methodological discussion.

**Setting:**

NHS Trusts in England.

**Methods:**

We established a research recruitment process with quality health (QH), administrators of the National Cancer Patient Experience Survey, after an amendment to a Section 251 approval (reference number ECC-8-05d-2011). NHS Trusts agreeing to implement the process were requested to send the details of 16–24-year-olds, identified by a relevant ICD-10 code indicating a cancer diagnosis within a specified time period to QH. QH sent study information and a consent-to-be-contacted form which allowed QH to send details to BRIGHTLIGHT, for BRIGHTLIGHT to contact the treating team confirming eligibility and for an interviewer from Ipsos MORI to contact them. Written consent was to be obtained at interview.

**Results:**

The method was implemented in 98 trusts; 75 supplied patient details. QH sent information to 441 young people, of whom 64 (15%) responded. Of these, 23 had already consented to participate. Adverse events were reported by 6 (1%) invitees: 4 were distressed because they did not have cancer, their details being submitted to QH due to incorrect hospital coding, and 1 young person was distressed about their diagnosis and requested no further contact and 1 young person found out they had cancer from the invitation.

**Conclusions:**

Application of Section 251 of the NHS Act (2006) to directly approach participants can facilitate recruitment to research projects where routinely collected NHS data are available to select eligible patients. The benefits of this method are that it requires fewer resources to recruit across multiple sites, and is quicker. Further information on the impact on bias and adverse event profile are required.

**Strengths and limitations of this study**

- This method could be applied to recruit to low-risk research.
- The increase in the availability of routinely collected data in the National Health Service (NHS) makes the method applicable to multiple indications.
- This method is cost-effective.
- This method may reduce the bias introduced by intermediaries in the recruitment process.
- The method is dependent on data accuracy in NHS Trust returns.

## Introduction

Despite national policies aimed to promote research in the National Health Service (NHS), a large number of research studies do not recruit to target or within the predicted time frame.^1^ This can have adverse impact on costs, the timeliness and relevance of the research questions and the significance of evidence generated.^2^ With increasing financial pressures on the NHS and reduced availability of funding for research, the impact of suboptimal recruitment to studies is evident; If recruitment has to be extended to reach the required sample size, the trial will cost more and take longer, delaying the use of the results in clinical practice. If trials become more expensive and take longer, fewer trials can be conducted overall with the limited funding and resources available.^3^ More recently, further attention has been given to increasing the value of research and reducing waste which includes increasing the efficiencies of recruitment and retention in studies.^4^ There is a substantial body of evidence around recruitment to clinical trials; however, the problems of recruiting to other types of study are also described.^5^ A previous study illustrated that less than a third (31%) of cohort studies based in the UK achieved their recruitment target^1^ and under-recruitment appears to be universal across country, study design and clinical area.^6^ ^7^

The role of the healthcare professionals is central to facilitating participation in research. However, an alternative role, as a barrier to recruitment, is beginning to be realised.^8^ ^9^ Professional gatekeeping or paternalism is not wholly understood, but its origins may lie within a sense of responsibility to protect patients from perceived harm.^9^ In the study by Borschmann *et al*,^8^ gatekeepers are described as basing referral to research on criteria other than those specified in the study protocol and making assumptions on suitability based on their estimate of the likelihood of the potential participant accepting their assessment of the benefit of the research and the predicted impact on their clinical relationship.

A further example of professional gatekeeping emerged during BRIGHTLIGHT, a National Institute for Health Research (NIHR)-funded cohort study, which aimed to recruit 2012 young people with cancer over 18 months. BRIGHTLIGHT was designed with the assistance of young people with cancer and in conjunction with healthcare professionals. It is the largest study conducted for teenagers and young adults with cancer and poses the question ‘Do specialist Cancer Services for teenagers and young adults add value?’. The study provided evidence for existing health policy that advocates specialists ‘age-appropriate’ care for young people. Healthcare models for this group have arisen without a substantial pre-existing research evidence base and the holistic multidisciplinary approach to care for teenagers and young adults (TYA) in the UK was becoming under increasing pressure and international scrutiny to provide evidence for effectiveness and costs. Critically, four key points around ‘age-appropriate’ specialist services remained unanswered. (1) What is specialist care for young people? (2) Does specialist care improve outcomes for young people with cancer? (3) What are the key components of specialist care? (4) How much does specialist care cost the NHS, young people and their families? The study is organised in four workstreams and six interrelated studies of which the longitudinal cohort constitutes the major component of the study (http://www.brightlightstudy.com).

BRIGHTLIGHT was designed around the ‘5A’ principles^9^ ^10^ and included a substantial period of feasibility work with patients and engagement with key stakeholders.^11^ The 5As principles set out a strategy to maximise opportunities for the young people with cancer to participate in research studies. However, the principles are relevant to other populations. Improved participation opportunities are possible when studies are ‘Appropriate’ in that the eligibility criterion, including age, is permissive of the inclusion of the population being studied, in this case, TYA. The age eligibility criterion of BRIGHTLIGHT was to include those aged 13–24 years at diagnosis in keeping with the service configurations that were being examined. The ‘Availability’ and ‘Access’ of the study is key to recruitment. BRIGHTLIGHT was conducted in the majority of NHS Trusts (over 100) in England and included all 13 principal (specialist) treatment centres for young people's cancer. The ‘Acceptability’ of the research question is also essential to successful inclusion. If studies are deemed irrelevant or poorly designed by healthcare professionals, they are unlikely to offer the study to patients and similarly if the questions are not relevant for patients or the study procedures are unpleasant or lengthy, refusal to participate is more likely. We designed BRIGHTLIGHT with young people and healthcare professionals, working together to optimise study design and outcomes.^10^ The fifth A, ‘Awareness’, relates to professional and patient awareness of a study and the importance of offering access. Prior to opening, we embarked on a nationwide ‘Awareness’ campaign that included professional and patient conferences, network managers and social media campaigns. Consequently, from inception, the study had national support and engagement from patients, research networks, healthcare professionals and relevant charitable bodies. Despite this level of engagement, recruitment to the study fell below that expected, requiring multiple protocol amendments to introduce changes aimed at facilitating study entry, a recruitment extension of 12 months and a reduction of the planned sample size with the acceptance of lower statistical power.

Screening logs were requested from each participating Trust to explore reasons for lower than anticipated recruitment. This was to rule out patient refusal as the primary cause. Our anticipated refusal rate had been 35% based on the previously published studies in this population;^12–14^ however, the screening logs showed an actual refusal rate of 22%. Further analysis of the screening logs illustrated that 42% of potentially eligible patients had not been approached regarding the study. Reasons for exclusion cited on the screening logs included many that were not protocol exclusion criteria.

The screening logs identified that young people appeared to have limited access to the study even in Principal Treatment Centres. We therefore sought alternative methods of recruitment that might reduce the need for healthcare professional input. Consultation with the BRIGHTLIGHT patient and public involvement group regarding access to research suggested support for receiving a direct invitation to participate in research.^9^ One such method was then identified that had been used routinely with patients in the NHS for several years, including young people with cancer.

Section 251 of the NHS Act (2006) in the UK enables the Secretary of State for Health to set aside the common law duty of confidentiality for defined medical purposes. It was recognised that some activities within the NHS, including research, required identifiable patient information, but obtaining consent to use this information was not always possible. Applications to access patient information are required to show due regard for the eight principles of the Data Protection Act (1998) and demonstrate that there is no other way of involving patients without this direct approach. We describe our experience of using Section 251 to identify and recruit potential research participants to a cohort study, and discuss its potential for use in a wider research context.

## Methods

### NHS patient experience surveys

There are a number of patient experience surveys administered annually in the NHS, which are managed by commercial research companies contracted to the NHS.^15^ Recruitment to the National Cancer Patient Experience Survey (NCPES) is coordinated by Quality Health (QH).^16^ Every year, over a 3-month period, patients who have been discharged following an in-patient or day case admission are identified through hospital Patient Administration Systems (PAS). Patients are eligible for inclusion if they are aged 16 or over and have a primary diagnosis of cancer indicated by the relevant ICD-10 code. Each NHS Trust has a designated Survey Lead who is responsible for checking the list of patients to ensure those who have died are not included and they have an appropriate ICD-10 code before submitting the data through secure data transfer systems to QH. A second level check for mortality is undertaken at QH before postal copies of the NCPES are sent to patients.^17^ This process has been implemented since 2010, with improvements in practice being introduced over time to ensure that patients are not inappropriately contacted, for example, ICD-10 codes are no longer allocated to patients until there is a confirmed diagnosis of cancer.

### Application of the method to BRIGHTLIGHT

**Figure 1:**
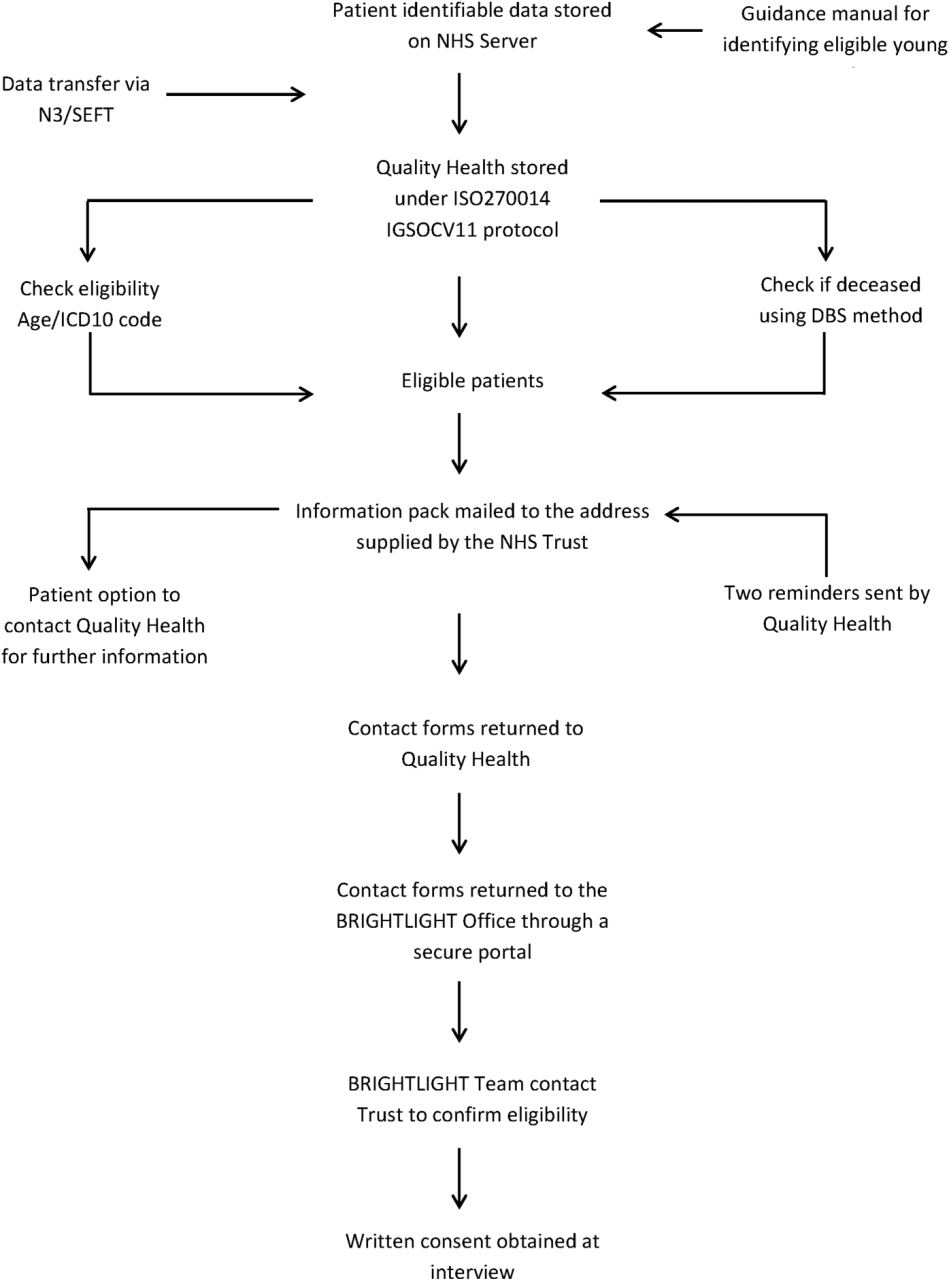
Schematic representation of the NCPES-based method for recruitment. DBS, Demographics Batch Service; NCPES, National Cancer Patient Experience Survey; NHS, National Health Service.

We worked with QH on an adapted version of the method used for the NCPES. A prior risk assessment for this process suggested that there would be minimal risk to patients but where low or medium risk was identified, processes could be implemented to protect their safety (table 1). BRIGHTLIGHT already had Section 251 approval in place to enable the Cancer Waits data set to be used to identify patients eligible for the cohort (ECC-8-05d-2011). An amendment was approved by the Health Research Authority (HRA) to allow patient details being transferred to QH without their consent (figure 1).

**Table 1:** Risk assessment for the NCPES-based recruitment method

Potential risk	Action to limit or resolve risk	Risk rating
Inappropriate patients are identified by NHS Trusts	Trusts to be given a guidance manual with clear criteria for selecting patients. Based on the NCPES—holding codes are no longer used, so patients given an ICD-10 code will have cancer	Low
Young people who have died are contacted to participate	Quality Health under take DBS checks to remove deceased patients	Low
Patient identifiable data from NHS Trust that is being used without patient consent being transferred incorrectly	Data transfer using the methods that exist for the NCPES, which Trusts are familiar with	Low
Contact forms being sent to incorrect address	Only the addresses provided by the NHS Trust will be used	Low
Young persons do not know they have cancer	BRIGHTLIGHT and Quality Health have advice lines for young people to contact if they have any concerns about the study. Links would be made to the clinical team to provide appropriate support	Low
Contact forms do not get returned by post	Follow-up letters to be sent on two occasions	Medium
Recruiting Trusts stop recruiting using standard methods because the NCPES-based method is viewed as a replacement	Letter to be sent to principal investigators explaining why this method is being used in conjunction with existing routes to recruitment	Medium
Young people are approached twice about the study	Covering letter with the information pack to state that if they have already been contacted/consented to BRIGHTLIGHT to return the letter in the envelop provided so they do not receive reminder letters	Medium
Young people have been transferred to another Trust, so it is not possible to confirm eligibility	The BRIGHTLIGHT team have a network of contacts across recruiting Trusts so they would identify the Trust the young person has been transferred to and contact the team there. If the identifying Trust does not know the location of where the young person has been transferred to, this information will be sought from the North West Knowledge Intelligence Team under the existing Section 251 approval	Low
Young people have been transferred to a Trust that is not recruiting to BRIGHTLIGHT	The BRIGHTLIGHT team would contact the Principal Treatment Centre to determine whether they would be able to confirm eligibility (from their MDT information). If eligibility can be confirmed, the young person will be invited to participate as detailed in the protocol. If eligibility cannot be confirmed, the BRIGHTLIGHT team will contact young people to explain the error and will offer them a place in the YAP, the BRIGHTLIGHT user group	Low*
Patients are found to be not eligible after the contact form has been returned and checks made with the clinical team by the BRIGHTLIGHT team	The BRIGHTLIGHT team will contact the young person to explain the error. If young people have a cancer diagnosis, they will be offered a place in the YAP	Low
Consent forms are not returned to the BRIGHTLIGHT team after the young person has been interviewed	Ipsos MORI field interviewers will receive additional training and will return the consent form with their contact records when the interview is complete	Low
A high attrition rate by young people recruited by this method	There is a dropout rate between recruitment and data collection at the first wave of 25%. Potentially there may be a higher rate of dropout if information has not been given face-to-face. There is insufficient evidence to quantify this and therefore the BRIGHTLIGHT team and Ipsos MORI will monitor dropout (and subsequent attrition at waves 2–5) in this group separately from the rest of the cohort	Low†

*If a Trust is not participating, then information required in the case report form may not be obtainable, so there will be the potential for missing data. This is anticipated as being low risk.

†Risk to data collection and not to young people.

DBS, Demographics Batch Service; NCPES, National Cancer Patient Experience Survey; YAP, Young Advisory Panel.

We prepared information about the study to send directly to young people to allow them to agree in principle to take part. The information pack contained a study leaflet and contact form with details of a freephone number if they required further information. The contact form was for young people to agree to their details being sent to the BRIGHTLIGHT team, for their healthcare team to be contacted (to double check for exclusion criteria) and for their details to be sent to Ipsos MORI, the research organisation undertaking the interviews, who would obtain signed consent at the time of interview. The exclusion criteria check was to identify if any young person were in prison, and if young people had advanced disease, to ensure they would still be alive at the time of interview.

The guidance sent to NHS Trusts requested details of patients aged 16–24 years with a new cancer diagnosis, indicated by the relevant ICD-10 codes. The same process of drawing and checking the sample used for the NCPES was applied. Four data extraction requests were planned for patients diagnosed between July and September, October, November and December 2014.

## Results

Principal investigators for the 104 Trusts which were open to recruitment were informed that this process was going to be implemented. No objections were raised. After gaining HRA and Research Ethics Committee approval, four Research and Development (R&D) departments objected to the amendment without specifying a reason, one R&D would not approve the amendment because the sample would be small and therefore they would be identifiable from young people's responses (the Trust was open to recruitment using existing mechanisms) and one R&D would not accept the Section 251 approval and insisted on the completion of numerous lengthy documents for an internal Information Governance review. This Trust was excluded from participating. QH therefore made sample requests to 98 NHS Trusts.

While 75 Trusts responded to the initial sample request for patient details, only 57 trusts supplied patient details by the fourth request. No data were submitted by 18 Trusts. Details of 441 young people were returned, of whom 64 (15%) agreed to be contacted by an interviewer (table 2). Of these, 23 had already consented to BRIGHTLIGHT through established mechanisms. Seven young people actively refused to be contacted further, 352 [young people did not respond] 5 were returned by Royal Mail as having moved, 3 were identified as having died and 7 patients were ineligible for the study. A further three young people could also not be included because information was not returned by their treating Trust to ensure that they were definitely eligible for participation.

**Table 2:** Summary of Trust involvement and data returned

	Trust involvement, n (%)	Sample returned, n	Response, n (%)
Sample 1: July to September	75 (76.5)	213	21 (9.9)
Sample 2: October	69 (70.4)	102	22 (21.6)
Sample 3: November	59 (60.2)	55	8 (14.5)
Sample 4: December	57 (58.2)	71	13 (18.3)
Total		441	15 (14.5)

A total of six young people/parents raised concerns with the BRIGHTLIGHT team and QH after receiving information. Four young people's details had been submitted based on having cancer when they did not have a QH's cancer diagnosis due to incorrect hospital coding, one young person found out through receiving information that he had cancer and one young person who was distressed about their cancer diagnosis did not want to receive any further information. All their healthcare teams were contacted to provide appropriate support and reassurance to young people.

## Discussion

We have tested a method for directly contacting patients to request participation in a low-risk research study which had broad inclusion criteria. It has potential for use by researchers in other fields. The uptake rate of 15% appears low and ranged from 10% to 22% between samples. Although low, young people are typically defined as a ‘hard to reach’ population for researchers and this method was implemented with no national advertising through NHS trusts or social media typically frequented by young people. The NHS Cancer Experience Patient Survey is accompanied by widespread advertising in trusts with a response rate of around 35% in young people rising to 60% for those aged 65–70 indicating that with appropriate publicity in place prior to recruitment will enhance the potential to recruit to studies by this method.

Although 35% of young people aged 16–24 years return the NCPES in our experience of working with young people, requesting information to be returned through the post has limited success. Previous work we have carried out with young people regarding optimising recruitment methods to research studies supported this: ‘If I was posted a letter to take part in a study I might not be as pro-active in replying and sending back’.^5^ It could be assumed that obtaining consent via electronic methods may have resulted in a higher return; however, we implemented this process rapidly as a ‘quick-fix’ remedy to ensure that the target sample size was achieved and time and resource constraints prevented us exploring other electronic methods for gaining young people's consent. Additional consenting methods more appropriate to the age group are worthy of consideration in future studies to maximise opportunities for young people to participate.

When we asked young people what types of research it would be appropriate to contact them by post, they agreed it would be acceptable for most non-drug study types and, if it was supported with dialogue with healthcare professionals, also for treatment-related studies.^9^ This supports work undertaken by the HRA patients' desire to have the option of choosing whether or not to take part in research.^18^ The NIHR launched the ‘Okay to ask’ campaign in 2014 encouraging patients to ask healthcare professionals about available research.^19^ However, recent evaluation of this campaign has shown that although 95% of patients felt it was important to ask about research, only 21% said they felt comfortable to do so.^20^ This method presents an opportunity to present available research to patients mitigating any barriers which may be perceived between patients and/or professionals about approaching the topic of research.

As discussed previously, the need for rapid implementation meant that there was limited advertising within Trusts, mostly relying on dialogue with the principal investigators and R&D departments. Formal letters to Chief Executives, advertising materials for relevant departments and links between the BRIGHTLIGHT team and Survey Leads, rather than QH, might have facilitated samples to be drawn from all Trusts to which requests were made. With this in mind, the total cost of implementation was £5500, equating to £86 per patient, slightly higher than the £50 NHS support cost that had been calculated for recruitment. Greater promotion of the method and its use in different populations might increase uptake rates and so reduce the cost per capita recruited.

The small number of young people who were contacted inappropriately indicates a limitation of this method. Some Trusts continue to use ICD-10 codes before diagnostic investigations are complete and some lists were returned to QH without the appropriate checks having been undertaken. By engaging a research organisation with extensive experience of this method, the incidence of error was small (1.4%) and having a robust mechanism in place to support those young people affected by errors ensured that any problems were addressed promptly with patients.

In conclusion, application of Section 251 of the NHS Act (2006) to directly approach participants can facilitate recruitment to research projects where routinely collected NHS data are available to identify eligible patients for the study. The benefits of this method are that it requires fewer resources to recruit across multiple sites and is quicker. The method may also overcome selection bias introduced by recruitment intermediaries; however, this is a matter that requires further exploration. If this method were to be applied in conjunction with widespread advertising as described above, it may be that the level of publicising within trusts would vary as would promotion to certain groups of patients to encourage participation when the invite arrives, and this may introduce additional bias. Overall, it is an approach that warrants consideration for other studies.
